# Non-Metabolic Functions of PKM2 Contribute to Cervical Cancer Cell Proliferation Induced by the HPV16 E7 Oncoprotein

**DOI:** 10.3390/v13030433

**Published:** 2021-03-08

**Authors:** Seoung-Ae Lee, Charles Ho, Madison Troxler, Chin-Yo Lin, Sang-Hyuk Chung

**Affiliations:** Center for Nuclear Receptors and Cell Signaling, Department of Biology and Biochemistry, University of Houston, Houston, TX 77204, USA; slee96@central.uh.edu (S.-A.L.); cho17@uh.edu (C.H.); mtroxler97@gmail.com (M.T.); clin23@central.uh.edu (C.-Y.L.)

**Keywords:** HPV16 E7, cervical cancer, PKM2, ML265, non-pyruvate kinase function

## Abstract

Pyruvate kinase M2 (PKM2) mainly catalyzes glycolysis, but it also exerts non-glycolytic functions in several cancers. While it has been shown to interact with the human papillomavirus 16 (HPV16) E7 oncoprotein, the functional significance of PKM2 in HPV-associated cervical cancer has been elusive. Here, we show that HPV16 E7 increased the expression of PKM2 in cervical cancer cells. TCGA data analyses revealed a higher level of PKM2 in HPV^+^ than HPV^−^ cervical cancers and a worse prognosis for patients with high PKM2 expression. Functionally, we demonstrate that shRNA-mediated PKM2 knockdown decreased the proliferation of HPV^+^ SiHa cervical cancer cells. PKM2 knockdown also inhibited the E7-induced proliferation of cervical cancer cells. ML265 activating the pyruvate kinase function of PKM2 inhibited cell cycle progression and colony formation. ML265 treatments decreased phosphorylation of PKM2 at the Y105 position that has been associated with non-glycolytic functions. On the contrary, HPV16 E7 increased the PKM2 phosphorylation. Our results indicate that E7 increases PKM2 expression and activates a non-glycolytic function of PKM2 to promote cervical cancer cell proliferation.

## 1. Introduction

While cervical cancer screening methods (i.e., the Pap test and HPV test) effectively prevent cervical cancer, they are not readily available to women in developing and underdeveloped countries. Cervical cancer is the third most common cancer and the third leading cause of cancer death among women worldwide [1]. High-risk human papillomavirus (HPV), in particular HPV16, is the major risk factor for this malignancy [2,3]. The HPV16 E7 oncoprotein promotes cervical cancer by interacting with cellular proteins crucial for the regulation of cell proliferation and survival [4].

The most notable HPV16 E7 targets are pRb, p107, and p130, which are commonly inactivated by DNA tumor viruses to promote S-phase entry [5]. While the expression of HPV16 E7 promotes cervical cancer in mice [6], the concurrent deletion of genes for pRb, p107, and p130 is insufficient to cause the malignancy [7]. These results indicate that other E7 targets also contribute to E7-induced cervical cancer. Degradation of tyrosine-protein phosphatase non-receptor type 14 (PTPN14) by HPV16 E7 is required for efficient immortalization of primary human keratinocytes [8]. HPV16 E7 interacts with and activates DNA-methyltransferase 1 (DNMT1), resulting in hypermethylation of anti-tumor immunity-related genes [9,10]. While it has been shown that HPV16 E7 interacts with pyruvate kinase M2 isoform (PKM2) [11], the functional significance of this interaction has not been characterized in cervical cancer.

PKM2 promotes glycolysis by catalyzing the transfer of a phosphoryl group from phosphoenolpyruvate to ADP, producing pyruvate and ATP in the cytoplasm [12]. While the other pyruvate kinase isoforms are constitutively active, the pyruvate kinase function of PKM2 is activated by several metabolites. For example, fructose-1,6-bisphosphate (FBP, glycolysis intermediate), phosphoribosylaminoimidazolesuccinocarboxamide (SAICAR, purine biosynthesis metabolite), and serine promote PKM2 tetramerization and thereby activate its pyruvate kinase function [13,14]. On the contrary, the activation of growth factor receptors and T cell receptor results in phosphorylation on S37 and Y105 and blocks PKM2 tetramerization [15,16]. The monomeric/dimeric form of PKM2 is associated with its protein kinase activity and ability to translocate to the nucleus [17,18,19]. Nuclear PKM2 interacts with Oct4 and promotes the growth of glioma [20]. EGFR activation promotes nuclear translocation of PKM2 and its interaction with β-catenin, resulting in glioma growth [21]. Hypoxia induces the interaction between PKM2 and HIF-1α, promoting tumor growth and angiogenesis in a pancreatic cancer model [22]. The dimeric form of PKM2 phosphorylates STAT3 and increases the proliferation of colorectal cancer cells [18].

In this study, we investigated the prognostic value of PKM2 in cervical cancer and its role in the E7-induced proliferation of cervical cancer cells using TCGA data and cell models. We show that HPV16 E7 increased PKM2 expression and a high level of PKM2 was associated with a poor prognosis for cervical cancer patients. We also show that both shRNA-mediated knockdown of PKM2 and pharmacological induction of PKM2 tetramerization (i.e., activation of the pyruvate kinase activity) suppressed the proliferation of cervical cancer cells. Our results support that HPV16 E7 activates the expression and non-glycolytic functions of PKM2 to promote cervical cancer growth.

## 2. Materials and Methods

### 2.1. Cells and Plasmids

Cells were purchased from ATCC and cultured in DMEM (Hyclone, Pittsburgh, PA, USA), supplemented with 10% fetal bovine serum (FBS, GenDepot, Katy, TX, USA) and gentamicin (50 µg/mL, Life Technologies, Carlsbad, CA, USA) at 37 °C in a humidified tissue culture incubator and under 5% CO_2_. Transduced cells were selected with puromycin (1 µg/mL) to generate stable cell lines. The pBABE-puro vector expressing HPV16 E7 was a generous gift from Dr. Lambert (McArdle Laboratory for Cancer Research, University of Wisconsin-Madison). Retroviral pGFP-V-RS vectors expressing PKM2 shRNAs (Cat. No. TG302462) and scrambled shRNA (Cat. No. TR30013) were purchased from OriGene (Rockville, MD, USA), and the PKM2 shRNA clone B was used for experiments. The pGEX2T-18E7 plasmid was a kind gift from Dr. Karl Munger (Tufts University School of Medicine, Boston, MA, USA). The pGEX4T1-45E7 plasmid was generated by inserting E7 cDNA obtained from MS751 cervical cancer cells into the pGEX4T1 vector. The sequence was verified by DNA sequencing.

### 2.2. Transfection and Transduction

Cells were transfected using Lipofectamine 3000 (Invitrogen, Carlsbad, CA, USA) according to the manufacturer’s protocol. Phoenix-AMPHO retroviral packaging cells were used to generate retroviral vectors. Collected viruses were concentrated using PEG-*it* Virus Precipitation Solution (System Biosciences, Mountain View, CA, USA). For transduction, 70–80% confluent target cells were incubated with the virus in DMEM supplemented with heat-inactivated FBS and 6 µg/mL of polybrene.

### 2.3. RT-PCR

Total RNA was extracted using TRIzol reagent (Thermo Fisher Scientific, Waltham, MA, USA) according to the manufacturer’s protocol. Oligo dT was used to generate cDNA, and PCR was carried out using primers for *PKM2* (5′-GGCTCGTGGTGATCTA GGCATTGA-3′ and 5′-CAGACTTGGTGAGGACGATTATGG-3′) and *GAPDH* (5′-AC CACAGTCCAT GCCATCAC-3′ and 5′-TCCACCACCCTGTTGCTGTA-3′).

### 2.4. Subcellular Fractionation and Chemical Cross-Linking

Cytoplasmic and nuclear proteins were isolated using the NE-PER Nuclear and Cytoplasmic Extraction Reagents kit (Thermo Fisher Scientific) according to the manufacturer’s instructions. Cells were treated with 1% paraformaldehyde for 7 min for cross-linking and then with 125 mM glycine for 5 min for quenching. Cells were lysed in Tris-free lysis buffer (50 mM HEPES, 150 mM NaCl, 1 mM EDTA, 1% NP-40, 0.1% sodium dodecyl sulfate, pH 7.4).

### 2.5. Western Blot Assay

Total cell extracts were obtained by lysing cells in RIPA buffer (50 mM Tris-HCl pH 8.0, 150 mM NaCl, 1% NP-40, 0.5% sodium deoxycholate, 0.1% sodium dodecyl sulfate) supplemented with protease and phosphatase inhibitors. Protein concentrations were measured using a Bio-Rad Protein Assay kit (Bio-Rad, Hercules, CA, USA). Proteins were resolved on SDS-polyacrylamide gels and transferred onto polyvinyl difluoride membranes (Amersham, Pittsburgh, PA, USA) using a Trans-Blot Turbo system (Bio-Rad). Membranes were incubated with primary antibodies against PKM2 (Cell Signaling Technology, Danvers, MA, USA; Cat. No. 4053), pY105-PKM2 (Cell Signaling Technology, Cat. No. 3827), HA tag (GeneTex, Irvine, CA, USA; Cat. No. GTX115044), HPV16 E7 (Santa Cruz Biotechnology, Dallas, TX, USA; Cat. No. sc-65711), actin (Santa Cruz Biotechnology, Cat. No. sc-8432), lamin A/C (Santa Cruz Biotechnology, Cat. No. sc-376248), or GAPDH (Santa Cruz Biotechnology, Cat. No. sc-32233) followed by incubation with horseradish peroxidase-conjugated anti-mouse (SA001-500) or anti-rabbit secondary antibody (SA002-500) from GenDepot. Chemiluminescent signal was captured using an Amersham Imager 600 system (GE Healthcare Bio-Sciences, Uppsala, Sweden).

### 2.6. Co-Immunoprecipitation and GST-Pull Down Assay

For co-immunoprecipitation assays, total cell extracts were incubated with an anti-HPV16 E7 antibody (Santa Cruz Biotechnology, Cat. No. sc-6981) at 4 °C. Immune complexes were recovered using protein A-agarose beads (GenDepot). For GST-pull down assays, bacteria were lysed in lysis buffer (150 mM NaCl, 50 mM Tris-HCl, pH7.5, 10 mM EDTA, 3 mg/mL lysozyme, 1% Triton X-100, and protease inhibitors), and GST fusion proteins were purified with glutathione agarose beads (Takara Bio, Mountain View, CA, USA) according to the manufacturer’s instruction. The resulting complexes were then incubated with cell lysates.

### 2.7. Cell Counting, Colony-Forming, and Cell Cycle Assay

For cell counting assay, cells were seeded in 24-well plates and subjected to trypan blue exclusion assays. For the colony-forming assay, 100 cells per well were seeded in 6-well plates and cultured for 2 weeks. Colonies were fixed in methanol, stained with 0.05% crystal violet for 20 min, and counted with NIH ImageJ software. For cell cycle analysis, cells were fixed in 70% ice-cold ethanol, and DNA was stained with propidium iodide (50 µg/mL) in the presence of RNase A (100 µg/mL). Processed cells were analyzed by a BD Accuri C6 Plus Flow Cytometer (BD Biosciences, San Jose, CA, USA).

### 2.8. TCGA Data Analyses

The Open Science Framework has processed RNA-sequencing data from The Cancer Genome Atlas (TCGA) using kallisto, an RNA-seq quantification program [23]. Transcripts per million (TPM) values were downloaded from its website (osf.io/gqrz9, accessed on 11 June 2020). Clinical data for the Cervical Squamous Cell Carcinoma and Endocervical Adenocarcinoma (CESC) patients from TCGA were obtained using the TCGAbiolinks (2.12.6) [24]. The Genotype-Tissue Expression (GTEx) normal cervix cohort data from the recount2 project [25] was transformed into TPM values using the human genome 38 gene lengths from kallisto (0.46.0). We used Welch’s two-sample unequal variances t-test, which is designed to address a disparity in the number of samples between test groups. Transcript ID ENST00000335181 was used to identify *PKM2* mRNA. Kaplan–Meier survival analysis was conducted using the survival (3.1-12) and survminer (0.4.6) packages.

## 3. Results

### 3.1. High PKM2 Expression Is Associated with Poor Prognosis in Cervical Cancer 

**Figure 1:**
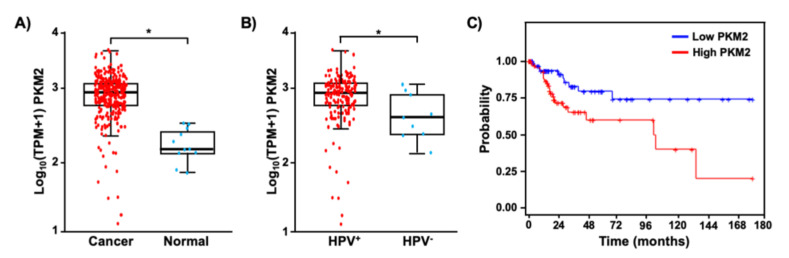
PKM2 is upregulated and has a prognostic value in cervical cancer. (**A**) TPM values of *PKM2* mRNA in cervical cancer tissues (*n* = 304) and normal cervical tissues (*n* = 11) are shown in the log scale. * *p* = 1.3 × 10^−6^ (Welch’s unequal variances *t*-test). (**B**) Cervical cancer tissues shown in A were grouped based on the HPV status. The level of *PKM2* mRNA was 2.1-fold higher in HPV^+^ cervical cancer (*n* = 169) than HPV^–^ cervical cancer (*n* = 9). * *p* = 0.05 (Welch’s unequal variances *t*-test). (**C**) Overall survival was worse in cervical cancer patients with high levels of PKM2 (red, *n* = 76) than those with low levels of PKM2 (blue, *n* = 75).

It has been shown that PKM2 is overexpressed in several types of tumors [26,27,28]. We first sought to determine whether PKM2 is also upregulated in cervical cancer. TCGA and GTEx data analysis showed that the median transcript level of PKM2 was 5.9-fold higher in cervical cancer tissues than in normal tissues (Figure 1A). Interestingly, its expression was significantly higher in HPV-positive cervical cancers than in HPV-negative cancers (Figure 1B). We then grouped patients by the top and bottom quartile of PKM2 expression and compared the overall survival probability of patients with high and low PKM2 levels (Figure 1C). Kaplan–Meier analysis showed that cervical cancer patients with high levels of PKM2 had worse overall survival than those with low levels of PKM2 (hazard ratio = 2.59, log-rank *p*-value = 0.01). These results suggest that PKM2 may play a role in the pathogenesis of HPV-induced cervical cancer.

### 3.2. HPV16 E7 Upregulates PKM2 in Cervical Cancer Cells

**Figure 2:**
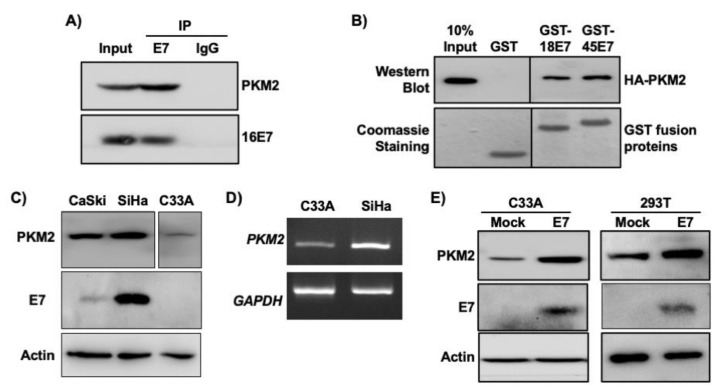
HPV16 E7 upregulates and interacts with PKM2 in cervical cancer cells. (**A**) HPV16 E7 interacts with PKM2 in SiHa cells. SiHa cell extracts (0.5 mg) were subject to co-immunoprecipitation using an anti-HPV16 E7 antibody. Normal IgG was used as a negative control. The input lane represents 50 μg of cell extracts. (**B**) GST proteins fused to HPV18 E7 (GST-18E7) and HPV45 E7 (GST-45E7) were incubated with 293T cell extracts overexpressing HA-tagged PKM2 (HA-PKM2). GST was used as a negative control. PKM2 was detected by western blot using an anti-HA antibody. GST fusion proteins were visualized by Coomassie blue staining. Intervening lanes were deleted and indicated by vertical lines. (**C**) PKM2 levels were higher in HPV^+^ than in HPV^−^ cervical cancer cells. Cell extracts were subject to western blot. Intervening lanes were deleted and indicated by vertical lines. (**D**) The levels of *PKM2* mRNA was higher in SiHa cells than C33A cells. Total RNA was subject to semi-quantitative RT-PCR. The number of PCR cycle was 25 for *PKM2* and 22 for *GAPDH*. (**E**) HPV16 E7 increased the level of PKM2. Cells were transfected with an empty (mock) or HPV16 E7-expressing plasmid. Cell extracts were subject to western blot. Actin was used as a loading control.

The interaction between E7 and PKM2 has previously been demonstrated in mouse fibroblasts [11,27]. In HPV16^+^ SiHa cervical cancer cell extracts, PKM2 was co-immunoprecipitated with HPV16 E7 by an E7 antibody, but not by a normal IgG control antibody (Figure 2A). To determine whether other high-risk HPV E7 oncoproteins also interact with PKM2, we used bacterially expressed HPV18 E7 and HPV45 E7 fused to GST. PKM2 was pulled down with both GST-E7 fusion proteins but not with GST (Figure 2B), indicating their interactions. We next investigated whether HPV status correlated with PKM2 levels in cervical cancer cell lines as in clinical samples shown in Figure 1B. PKM2 protein levels were higher in HPV16-positive CaSki and SiHa cervical cancer cells than in HPV-negative C33A cells (Figure 2C). *PKM2* mRNA levels were also higher in SiHa than in C33A cells (Figure 2D). Transient overexpression of HPV16 E7 increased PKM2 expression in C33A cells and 293T non-cervical cells (Figure 2E). These results reveal that HPV16 E7 not only interacts with PKM2 but also upregulates its expression.

### 3.3. PKM2 Contributes to E7-Induced Cervical Cancer Cell Proliferation

**Figure 3:**
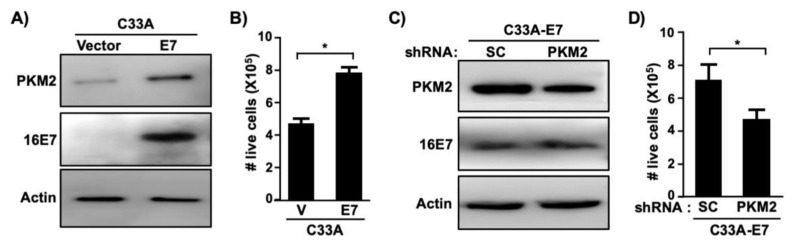
HPV16 E7-induced proliferation of cervical cancer cells depend on PKM2. (**A**) C33A cells stably expressing HPV16 E7 or empty vector were generated using retroviral vectors. Cell extracts were analyzed with western blot. (**B**) Cells described in A were seeded in 24-well plates (20,000 cells/well) and counted after 5 days. Results from five independent experiments are shown as mean ± S.E.M. V, vector. * *p* = 0.005 (two-sided Student’s *t*-test). (**C**) C33A-E7 cells were transduced with retrovirus expressing scrambled (SC) shRNA or PKM2 shRNA. Total cell extracts were subject to western blot. (**D**) Cells described in C were subject to cell counting assay, as described in B. Results from three independent experiments are shown as mean ± S.E.M. * *p* = 0.01 (two-sided Student’s *t*-test).

**Figure 4:**
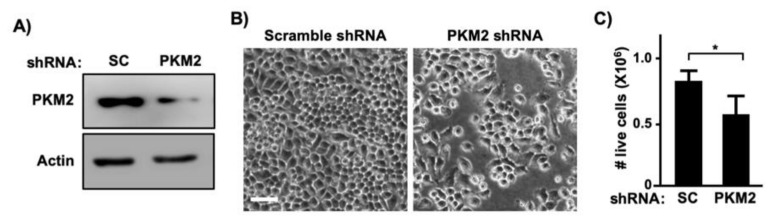
PKM2 is required for proliferation of cervical cancer cells. (**A**) SiHa cells were transiently transfected with scrambled (SC) shRNA or PKM2 shRNA vector. Total cell extracts were analyzed with western blot. (**B**) SiHa cells were transiently transfected as described in A and photographed 5 days later. Note that cells transfected with an PKM2 shRNA plasmid were sub-confluent. Scale bar, 100 µm. (**C**) SiHa cells were transiently transfected as described in A and counted 5 days later. Data are presented as mean *±* S.E.M. (*n* = 3). * *p* = 0.01 (two-sided Student’s *t*-test).

To determine whether PKM2 is required for E7-induced cell proliferation, we createdC33A cells stably expressing HPV16 E7 (Figure 3A). Consistent with results shown in Figure 2E, PKM2 levels were elevated in C33A-E7 cells as compared to C33A-vector control cells (Figure 3A). Exogenous expression of E7 significantly increased the proliferation of C33A cells (Figure 3B). To determine whether PKM2 contributes to this E7-induced phenotype, we transduced C33A-E7 cells with retrovirus expressing PKM2 shRNA. PKM2 expression was reduced in PKM2 shRNA-transduced cells as compared to scrambled shRNA-transduced cells (Figure 3C). Knockdown of PKM2 did not affect E7 expression but resulted in a significant reduction in the cell number (Figure 3D). To determine whether PKM2 is crucial for the proliferation of HPV-positive cervical cancer cells, we knocked down PKM2 in SiHa cells by transient transfection (Figure 4A). Unlike scrambled shRNA-transfected cells, SiHa cells transfected with PKM2 shRNA did not become fully confluent at the experimental endpoint (Figure 4B). A cell counting assay also showed a significant reduction in the number of SiHa cells transfected with PKM2 shRNA (Figure 4C). These findings provide evidence that PKM2 contributes to the E7-induced proliferation of cervical cancer cells.

### 3.4. ML265, a PKM2 Activator, Inhibits the Proliferation of Cervical Cancer Cells

**Figure 5:**
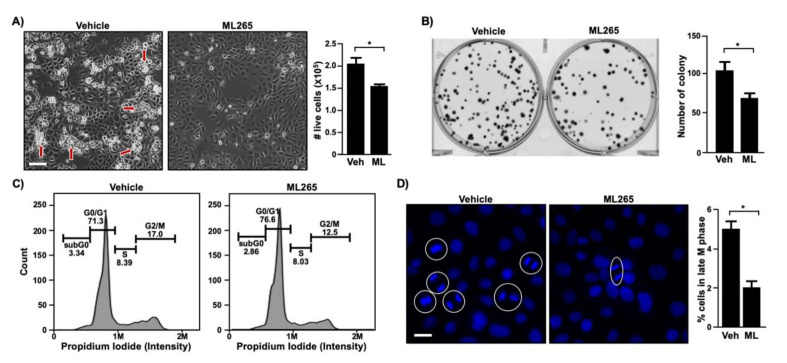
ML265 inhibits cell cycle progression and colony formation in cervical cancer cells. (**A**) SiHa cells were seeded in 24-well plates (20,000/well) and treated with vehicle (veh) and 40 μm of ML265 (ML) for 5 days. The left panel shows photographed cells (scale bar, 100 µm). Red arrows point to cells growing on top of other cells. The right panel shows cell counting results as mean *±* S.E.M. (*n* = 3). * *p* = 0.03 (two-sided Student’s *t*-test). (**B**) SiHa cells were seeded in 6-well plates (100 cells/well) and treated with 40 μm of ML265 for 2 weeks. Cells were stained with crystal violet and photographed (*left panel*). Quantified results (*right panel*) are shown as mean ± S.E.M. (*n* = 3). * *p* = 0.04 (two-sided Student’s *t*-test). (**C**) SiHa cells were treated with ML265 (40 µm) for 24 h. Cell cycle was analyzed with flow cytometry. Shown are representative results from two independent experiments with triplicate samples (*p* < 0.05, Student’s paired *t*-test). At least 10,000 cells per sample were analyzed. (**D**) SiHa cells were treated with ML265 (40 µm) for 24 h and stained with Hoechst 33342 to visualize DNA (blue). Random fields of view were photographed, and mitotic cells were quantified. The *left panel* shows the representative images of each group (scale bar, 50 µm). Cells in anaphase and telophase are circled, and each circle is counted as one cell. More than 1,000 cells per sample were analyzed. Quantification results are shown in the *right panel* as mean ± S.E.M (*n* = 3). * *p* = 0.003 (two-sided Student’s *t*-test).

To determine whether the activation of the PKM2 pyruvate kinase function promotes cervical cancer cell proliferation, we treated SiHa cells with the small molecule compound ML265 that is known to promote the formation of PKM2 tetramer [29]. Vehicle-treated SiHa cells were confluent and formed multi-layered cell clumps, but ML265-treated cells were sub-confluent and barely made multi-layers (Figure 5A, left panel). Concordantly, cell numbers significantly decreased in the ML265-treated group as compared to the vehicle-treated control group (Figure 5A, right panel). ML265 treatment also reduced the ability of SiHa cells to form colonies (Figure 5B). Cell cycle analysis showed the accumulation of SiHa cells in the G0/G1 phase and decreased proportion in the G2/M phase in the ML265-treated group as compared to the vehicle group (Figure 5C). While the differences were only 4.5–6%, results were reproducible, and the difference was statistically significant. Unbiased nuclear image analysis for cells in anaphase and telophase also showed that ML265 treatment significantly decreased the percentage of cells in the late M phase of the cell cycle (Figure 5D). These results demonstrate that ML265 prevented cervical cancer cell proliferation by blocking cell cycle progression.

### 3.5. ML265 Decreases a Phosphorylated Form of PKM2

**Figure 6:**
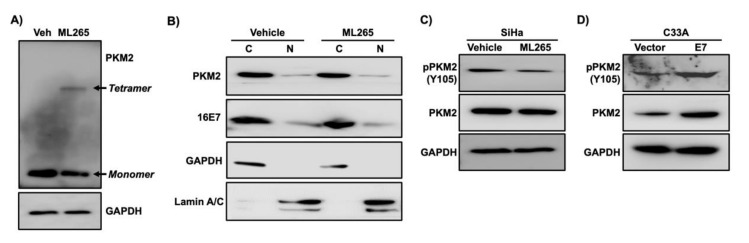
ML265 decreases the phosphorylation of PKM2 at the Y105 position. (**A**) SiHa cells were treated with ML265 (40 µm) or vehicle (veh) for 24 h and then treated with paraformaldehyde for cross-linking. Total cell extracts were analyzed with western blot. GAPDH was used as a loading control. Note that ML265 decreased monomer and increased tetramer. (**B**) SiHa cells were treated with vehicle and ML265 for 24 h. Cells were nearly confluent at the endpoint. The cytoplasmic fraction (C) and the nuclear fraction (N) were analyzed with western blot. GAPDH and lamin A/C were used as a cytoplasmic and nuclear fraction marker, respectively. (**C**) SiHa cells were treated with ML265 for 24 h. Total cell extracts were subject to western blot analysis. (**D**) Total cell extracts from C33A-vector and C33A-E7 cells described in Figure 3A were analyzed with western blot. Note that both pY105-PKM2 and total PKM2 were increased in C33A-E7 cells compared to the control cells.

In chemical cross-linking protein interaction experiments, the tetrameric form of PKM2 was detected in ML265-treated SiHa cells but not in vehicle-treated cells (Figure 6A). ML265 decreased the level of monomeric PKM2 (Figure 6A). These results confirmed that ML265 promoted the tetramerization of PKM2 in SiHa cervical cancer cells. It has been shown that oncogenic signaling inhibits PKM2 tetramerization and promotes nuclear translocation of monomeric PKM2, which are responsible for the increased proliferation of cancer cells [30,31]. We were unable to detect nuclear PKM2 in sub-confluent and confluent SiHa cells by indirect immunofluorescence assay [32]. However, using biochemical subcellular fractionation assay, we detected PKM2 in the nuclear fraction of SiHa cells (Figure 6B). Only a tiny fraction of PKM2 was in the nucleus. It should be noted that 10% of the total cytoplasmic fraction and 40% of the entire nuclear fraction were analyzed with western blot (i.e., the actual difference in the amount of PKM2 in the cytoplasmic and nuclear fraction is greater than what is shown in the figure). Notably, there was no appreciable difference in the level of PKM2 in the nuclear fraction between vehicle-treated and ML265-treated SiHa cells (Figure 6B), indicating that ML265 did not promote nuclear translocation of PKM2. Another non-glycolytic function is associated with phosphorylation of PKM2. The activation of Ras and ErbB2 phosphorylates PKM2 at the Y105 residue in the cytoplasm, which inhibits PKM2 tetramerization [33,34,35]. We found that ML265 reduced phosphorylation of PKM2 at Y105 (pY105-PKM2) in SiHa cells without affecting the total PKM2 level (Figure 6C). This effect was dose-dependent and reached the plateau at 40 µm (Supplementary Figure S1). On the contrary, the level of pY105-PKM2 increased in C33A-E7 cells compared to C33A-vector control cells (Figure 6D), indicating that HPV16 E7 increased phosphorylation of PKM2. Our findings indicate that ML265 inhibited the non-metabolic activity of PKM2 required for the proliferation of cervical cancer cells.

## 4. Discussion

Even though HPV16 E7 interacts with several dozens of cellular proteins, not all of them have been investigated for the relevance to cervical carcinogenesis [36,37]. Similar to several other cancers, PKM2 is upregulated in cervical cancer (Figure 1A). High levels of PKM2 are associated with poor prognosis in cervical cancer patients (Figure 1C). PKM2 knockdown inhibited the proliferation of SiHa cervical cancer cells (Figure 4). These results are consistent with that high PKM2 is associated with poor responses of locally advanced cervical cancer to radiation therapy and PKM2 knockdown sensitizes SiHa cervical cancer cells to radiation in vitro [38].

In this study, we demonstrated that PKM2 is required for HPV16 E7-induced cervical cancer cell proliferation to the fullest extent (Figure 3 and Figure 4). HPV16 E7 expression also increased the phosphorylated form of PKM2 (pY105-PKM2; Figure 6D), which does not form tetramers [35]. HPV16 E7 has been shown to inhibit PKM2 tetramerization and decrease the glycolytic flux rate in mouse fibroblasts [31,39]. These results suggest that E7 oncoprotein is an inhibitor of the pyruvate kinase function of PKM2, which is the action of tetramers [12]. Phosphorylated PKM2 is associated with non-pyruvate kinase functions and protumorigenic activities [35,40,41]. For example, pY105-PKM2 promotes cell proliferation and colony formation in anaplastic large cell lymphoma cells [40]. Inhibiting the phosphorylation of PKM2 at Y105 suppresses the proliferation and migration of lung cancer cells [41]. As expected, treatments of cervical cancer cells with ML265 increased PKM2 tetramerization (Figure 6A), indicative of activation of its pyruvate kinase function. However, it did not increase the nuclear localization of PKM2 (Figure 6B). It was surprising that the majority of E7 was present in the cytoplasmic fraction (Figure 6B). It may be related to our experimental condition that cells were nearly confluent. It has been shown that HPV16 E7 translocates to the cytoplasm when cells become confluent [42]. ML265 decreased the level of pY105-PKM2 and inhibited cell cycle progression and colony formation in SiHa cells (Figure 5 and Figure 6C). Based on these observations, it appears that HPV16 E7 activates non-glycolytic functions of PKM2 by increasing monomeric PKM2, which is achieved by binding and disrupting PKM2 tetramerization. Our results support that non-glycolytic activities of PKM2 are required for the E7-induced proliferation of cervical cancer cells. However, it should be noted that our results do not necessarily exclude the possibility that the glycolytic function of PKM2 is also necessary for E7-induced cell proliferation. As E7 upregulated PKM2 expression, at least in our experimental conditions (Figure 2C–E), E7 may increase both glycolytic and non-glycolytic function of PKM2.

HPV16 E7 increased PKM2 levels in C33A cervical cancer cells and 293T human kidney cells (Figure 2E). The pRb pathway is inactive in these cells because 293T cells express human adenovirus E1A and simian virus 40 large T oncoproteins and the *RB1* gene is mutated in C33A cells [5,43]. These results indicate that E7-induced upregulation of PKM2 was not due to the inactivation of pRb. PKM2 expression was not always elevated in HPV-positive cervical cancers (Figure 1B). HPV16 E7 has not increased PKM2 levels in NIH3T3 mouse fibroblasts [39]. These results suggest that E7-mediated PKM2 upregulation is context-dependent.

In summary, we showed that the HPV16 E7 oncoprotein not only interacts with PKM2 but also upregulates its expression. Our results and other published observations support that E7 activates the non-glycolytic function of PKM2 by inhibiting its tetramerization. Our results further support that this E7 activity is required for the proliferation of cervical cancer cells.

## Data Availability

Publicly available datasets were analyzed in this study. This data can be found here: osf.io/gqrz9.

## Supplementary Materials

The following are available online at https://www.mdpi.com/1999-4915/13/3/433/s1, Figure S1: ML265 inhibits PKM2 phosphorylation in a dose-dependent manner.
