# Supplementary material for: Non-Metabolic Functions of PKM2 Contribute to Cervical Cancer Cell Proliferation Induced by the HPV16 E7 Oncoprotein
Source: Viruses. 2021 Mar 8;13(3):433. doi: 10.3390/v13030433 (PMC8001101; doi:10.3390/v13030433)
Supplement: Supplementary file 1 [file viruses-13-00433-s001.pdf]

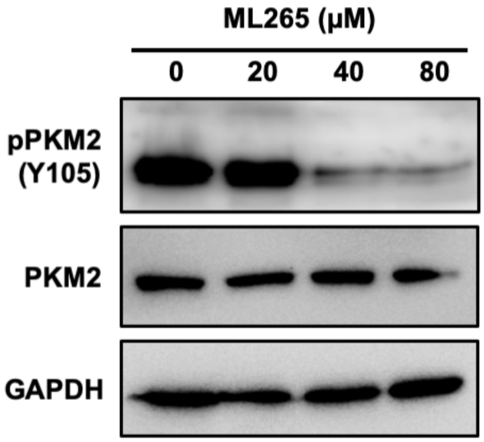

Supplementary Figure 1. ML265 inhibits PKM2 phosphorylation in a dose-dependent manner. SiHa cells were treated with ML265 for 24 hours. Cell extracts were subjected to western blot. Note that PKM2 phosphorylation was maximally inhibited at 40 μM.
